# Local Synchronization of Resting-State Dynamics Encodes Gray’s Trait Anxiety

**DOI:** 10.1371/journal.pone.0058336

**Published:** 2013-03-08

**Authors:** Tim Hahn, Thomas Dresler, Martin Pyka, Karolien Notebaert, Andreas J. Fallgatter

**Affiliations:** 1 Department of Cognitive Psychology II, Johann Wolfgang Goethe University, Frankfurt am Main, Germany; 2 Department of Psychiatry, Psychosomatics and Psychotherapy, University of Würzburg, Würzburg, Germany; 3 Department of Psychiatry and Psychotherapy, University of Tübingen, Tübingen, Germany; 4 Department of Psychiatry and Psychotherapy, University of Marburg, Marburg, Germany; 5 Research Center of Marketing and Consumer Science, Katholieke Universiteit Leuven, Leuven, Belgium; Institute of Psychology, Chinese Academy of Sciences, China

## Abstract

The Behavioral Inhibition System (BIS) as defined within the Reinforcement Sensitivity Theory (RST) modulates reactions to stimuli indicating aversive events. Gray’s trait Anxiety determines the extent to which stimuli activate the BIS. While studies have identified the amygdala-septo-hippocampal circuit as the key-neural substrate of this system in recent years and measures of resting-state dynamics such as randomness and local synchronization of spontaneous BOLD fluctuations have recently been linked to personality traits, the relation between resting-state dynamics and the BIS remains unexplored. In the present study, we thus examined the local synchronization of spontaneous fMRI BOLD fluctuations as measured by Regional Homogeneity (ReHo) in the hippocampus and the amygdala in twenty-seven healthy subjects. Correlation analyses showed that Gray’s trait Anxiety was significantly associated with mean ReHo in both the amygdala and the hippocampus. Specifically, Gray’s trait Anxiety explained 23% and 17% of resting-state ReHo variance in the left amygdala and the left hippocampus, respectively. In summary, we found individual differences in Gray’s trait Anxiety to be associated with ReHo in areas previously associated with BIS functioning. Specifically, higher ReHo in resting-state neural dynamics corresponded to lower sensitivity to punishment scores both in the amygdala and the hippocampus. These findings corroborate and extend recent findings relating resting-state dynamics and personality while providing first evidence linking properties of resting-state fluctuations to Gray’s BIS.

## Introduction

As a theory of personality firmly rooted in neurobiology, Gray’s Reinforcement Sensitivity Theory (RST; [1]) has grown into one of the most influential theories of personality, strongly influencing research in a number of disciplines including psychology, pharmacology, animal research, and neuroscience [2]. The RST postulates behavior to be mediated by the activity of three motivational systems: the Fight-Flight System, the Behavioral Approach System (BAS), and the Behavioral Inhibition System (BIS). The latter is triggered by signals of punishment or non-reward, which then leads to an inhibition of motor-activity and increased levels of arousal and attention (for a partially revised version of the theory, see [3]). Defined to reflect trait sensitivity to punishment, Gray’s trait Anxiety determines the extent to which aversive stimuli activate the BIS [4].

Clinically, heightened trait Anxiety (heightened BIS responsiveness) has been linked to emotional states of anxiety, subjectively experienced as worry and rumination, and a sense of possible danger/loss. Also, a number of mental disorders including depression, anxiety disorders, and psychosomatic illnesses have been associated with extreme BIS responsiveness [2].

To date, only few neuroimaging studies directly shed light on the neural underpinnings of Gray’s trait Anxiety: Barros-Loscertales et al. [5] were able to show that increased grey matter volume in the amygdala and the hippocampus was associated with increased trait sensitivity to punishment scores. Confirming this evidence in large sample, Cherbuin et al. [6] showed an association between a measure of BIS responsiveness and hippocampal volume while failing to find such an association with amygdala volume. Similarly, Hahn et al. [7] showed that variance in functional connectivity between the amygdala and the hippocampus during monetary loss anticipation can in part be attributed to individual differences in Gray’s trait Anxiety. This provides evidence that Gray’s trait Anxiety is associated with the functional dynamics of the amygdala-septo-hippocampal (SHS)-circuit during loss anticipation, which thereby displays a core property of the BIS. Unlike these approaches, further evidence is only indirect, showing higher amygdala and hippocampus activity following fearful faces and aversive pictures in subjects scoring higher on measures such as state or trait anxiety [8]–[10].

More recently, it has been shown that resting-state randomness is associated with Gray’s Sensitivity to Reward [11]. Similarly, Wei et al. [12] showed an association between Regional Homogeneity (ReHo; [13]) – a measure of local synchronization in resting-state BOLD fluctuations – and personality: Both neuroticism and extraversion traits were negatively correlated with ReHo in a distributed network of brain regions.

While this evidence clearly links resting-state fluctuations to personality traits, resting-state dynamics potentially associated with the BIS have not yet been explored. Here, we therefore investigate associations between Gray’s trait Anxiety and resting-state temporal dynamics of the amygdala-SHS circuit. Specifically, we explore the associations of the BIS with local resting-state synchronization as measured by ReHo in the amygdala and the hippocampus.

## Materials and Methods

### 1. Participants

Twenty-eight healthy subjects participated in the present study. One subject had to be excluded due to excessive head motion (>1.5 mm/degree in any direction). The remaining sample consisted of 14 females and 13 males with a mean age of 25.5 years (SD = 3.38). All were recruited from the local community through advertisements. The participants 1. reported no first-degree relative with a neurologic or mental disorder, 2. had no history of dependence on illicit drugs and alcohol, 3. were currently not taking any psychotropic medication, and 4. had no sensorimotor deficits or other neurological disorders. Written informed consent was obtained after detailed explanation of the study protocol. The study was approved by the Ethics Committee of the University of Würzburg, and all procedures involved were in accordance with the latest version (sixth revision) of the Declaration of Helsinki.

### 2. Psychometric Testing

All participants completed the Sensitivity to Punishment and Sensitivity to Reward Questionnaire (SPSRQ; [14]) in its German version (Hewig, J. and Hagemann, D. (2002). Der SPSR-Fragebogen von Torrubia, Avila, Molto und Caseras – unpublished German translation. University of Trier; personal communication). The SPSRQ is a 48-item self-report measure of Gray’s trait Anxiety and impulsivity dimensions. It consists of two scales, representing *sensitivity to reward* (SR) to measure impulsivity and *sensitivity to punishment* (SP) to measure anxiety. They are particularly designed to measure Gray’s concepts by linking SR to the BAS and SP to the BIS. The two scales show retest reliabilities of 0.87 and 0.89 for the reward and the punishment scale, respectively. In accordance with Gray’s theory, orthogonality of the scales has been confirmed, while good construct validity has also been shown [15].

### 3. Resting-state fMRI Acquisition

All subjects were scanned for 5 minutes. No specific instructions were given except to relax and hold still. Head movements were minimized by using a cushioned head fixation device. Imaging was performed using a 1.5 T Siemens Magnetom Avanto TIM-system MRI scanner (Siemens, Erlangen, Germany) equipped with a standard 12 channel head coil. In a single session, twenty-four 4-mm-thick, interleaved axial slices (in-plane resolution: 3.28 x 3.28 mm) oriented at the AC-PC transverse plane were acquired with 1 mm interslice gap, using a T2*-sensitive single-shot EPI sequence with following parameters: repetition time (TR; 2000 ms), echo time (TE; 40 ms), flip angle (90°), matrix (64×64), field of view (FOV; 210×210 mm^2^), and number of volumes (150). The first six volumes were discarded to account for magnetization saturation effects.

### 4. fMRI Resting-state Parameters

All analyses were conducted using the REST toolbox [16] with the Data Processing Assistant for Resting-State fMRI (DPARSFA; V2.1) with default parameters. First resting-state data was slice time corrected and head motion correction was applied. After head-motion correction, the fMRI images were normalized to the Montreal Neurological Institute (MNI) template. After preprocessing, the time series for each voxel was detrended and bandpass-filtered (0.01–0.08 Hz) to reduce low-frequency drift and physiological high frequency respiratory and cardiac noise. Individual ReHo maps were generated by calculating Kendall’s coefficient, i.e. concordance of the time series of a given voxel with those of its nearest neighbors (26 voxels) in a voxel-wise analysis. The resulting ReHo maps were then spatially smoothed with a Gaussian kernel of 6×6×6 mm full-width at half-maximum.

Based on the considerations concerning the BIS outlined above and in accordance with previous work [7], we focus all analyses on the amygdala and the hippocampus. ReHo was calculated for each voxel in the amygdala and the hippocampus. Both regions of interest (ROIs) were defined using voxel masks from the AAL atlas as implemented in SPM [17].

### 5. Second-level Correlation Analyses

We correlated (Pearson correlation) SP scores with ReHo mean over the amygdala and the hippocampus, respectively. In the additional an exploratory voxel-wise whole-brain analysis correlating ReHo at each voxel with the psychometric score, False Discovery Rate (FDR) correction as implemented in SPM5 was applied with an α-level of 5%.

## Results

**Figure 1:**
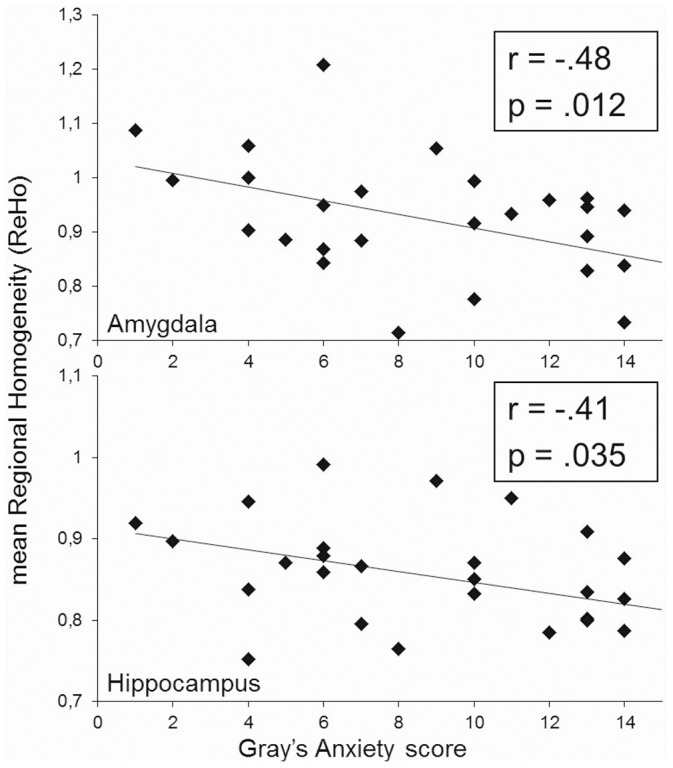
Association between Gray’s trait Anxiety (SP) scores and mean Regional Homogeneity (ReHo) over the amygdala (upper panel) and the hippocampus (lower panel).

Correlation analyses showed that SP-scores were significantly associated with ReHo in both the amygdala (r = −.48; p<.012) and the hippocampus (r = −.41; p<.035, Figure 1). SP scores explained 23% and 17% of ReHo variance in the amygdala and the hippocampus, respectively. Further analyses considering the hemispheres separately showed similar results for the left (r = −.48; p<.012) and right amygdala (r = −.39; p<.046) and the left hippocampus (r = −.44; p<.022), while revealing a trend for the right hippocampus (r = −.34; p<.087).

ReHo was not significantly associated with SR-scores for either ROI (p>.15). In addition, a whole-brain analysis correlating ReHo at each voxel with the psychometric scores yielded no significant results.

## Discussion

In this study, we explored the association between local synchronization of resting-state dynamics as measured by ReHo and Gray’s trait Anxiety as defined by the RST. We found individual differences in Gray’s trait Anxiety to be associated with ReHo in areas previously associated with BIS functioning, namely the amygdala and the hippocampus. Specifically, higher ReHo in resting-state neural dynamics in both regions corresponded to lower sensitivity to punishment scores. To our knowledge, this constitutes the first evidence linking properties of resting-state fluctuations to the BIS.

From these findings, the question arises, how the neurophysiological underpinnings of ReHo are associated with Gray’s trait Anxiety. Recently, it has been speculated that ReHo mirrors the degree of coherence of spontaneous neural activity [18]. Following this line of thought, individuals with higher scores in Gray’s trait Anxiety would show less coherent neural firing during rest than participants with lower scores. While this idea has been articulated previously [11], research directly linking neuronal coherence at rest to personality is missing. Supporting the idea, however, strong evidence from electroencephalography (EEG) suggests that resting-state neural activity generally influences task-related neural responses to stimulation: a strong association between spontaneous oscillations (i.e. pre-stimulus alpha-band activity) and the amplitude and shape of task-related EEG-responses has been shown ([19], [20]; for a review of the relation between background and task-related activity, see [21]). From this it can be argued that neural dynamics at rest impose constraints on the range of possible neural responses to stimulation. In other words, the properties of resting-state dynamics in a certain region appear to define the extent to which this area can respond to internal or external input (see Mennes et al., [22], for a similar view). In structures known to be associated with personality as investigated in our study, this might effectively constrain trait-related behaviors (for a similar argument relating to the randomness of resting-state dynamics, see [11]). Supporting this view, numerous studies have shown relationships between oscillations in the EEG and personality traits (for a recent overview, see [23]; for a comprehensive review of rest-stimulus interaction in general, see [24]). If ReHo captures relevant information related to resting-state neural dynamics, it should thus be associated with personality-related neural activation and eventually with personality.

In addition to the discussion of potential neurophysiological mechanisms underlying personality traits in general, interpreting the current results in the context of Gray’s RST is of interest: Basically, Gray’s trait Anxiety – defined to reflect trait sensitivity to punishment – determines the extent to which stimuli activate the BIS [4]. If, as outlined above, neural dynamics at rest do indeed impose constraints on the range of possible neural responses to stimulation, resting-state randomness might directly determine the extent to which stimuli can activate the neural underpinnings of the BIS. Thus, ReHo might more directly mirror Gray’s concept of BIS responsiveness. From this point of view, task-related activation during the anticipation of punishment might be considered subsequent to properties of underlying resting-state dynamics. Taking into account the positive correlation between SP-scores and loss-related functional amygdala-hippocampus connectivity [7] and our results showing a negative association between ReHo and Gray’s trait Anxiety, it can be speculated that lower resting-state ReHo also ought to entail stronger activation during the anticipation of punishment. Whether such a simple relationship between the amplitude of task-related activation holds or if more complex, potentially non-linear associations with other characteristics of the activation exist must be determined in future studies. A study investigating the relationship between resting-state dynamics and subsequent task-related activation might shed light on this issue. To fully characterize the complex relationship between resting-state dynamics and task-related activation, however, extensive further studies are needed.

In summary, we provide evidence for an association between individual differences in Gray’s trait Anxiety and ReHo in areas previously associated with Gray’s BIS. Specifically, higher ReHo corresponded to lower SP-scores. Thus, ReHo appears to link Gray’s concept of BIS responsiveness and neural activation during the anticipation of punishment. Generally, our results corroborate and extend recent findings linking resting-state dynamics and personality.
